# Comparison of the Application Value of Transthoracic Echocardiography in Diagnosing Patent Foramen Ovale Under Different States of Stimulation: A Retrospective Study

**DOI:** 10.1002/clc.24319

**Published:** 2024-08-07

**Authors:** Jianwei Shi, Haijuan Gu, Wenjun Fan, Jiesheng Xia, Huanhuan Gu

**Affiliations:** ^1^ Department of Ultrasound Haimen District People's Hospital Nantong China; ^2^ Department of Cardiology Haimen District People's Hospital Nantong China

**Keywords:** contrast‐enhanced transthoracic echocardiography (cTEE), diagnosis, patent foramen ovale (PFO), Valsalva maneuver

## Abstract

**Objective:**

This study aims to evaluate the application value of contrast‐enhanced transthoracic echocardiography (cTEE) in the diagnosis of patent foramen ovale (PFO) under different states of stimulation, with the goal of enhancing the accuracy and efficiency of PFO diagnosis.

**Methods:**

This research consecutively enrolled patients suspected of having PFO from October 2022 to February 2024, presenting primary clinical symptoms such as unexplained syncope, headache, dizziness, and stroke. Patients underwent standard transthoracic echocardiography (TTE) and cTEE under three different states of stimulation (resting state, coughing, and Valsalva maneuver). Based on the presence of microbubbles in the left heart and their initial appearance time, patients were classified into PFO and control groups, with further diagnostic confirmation via transesophageal echocardiography (TEE) or foramen ovale closure procedures.

**Results:**

The study results revealed significant differences between the PFO and control groups regarding age (*p* = 0.034) and headache symptoms (*p* = 0.001). In the PFO group, TTE showed a higher positivity rate both at rest and during coughing, highlighting the association between PFO and specific clinical symptoms. The number of microbubbles observed during TTE increased significantly under various stimulation states, particularly during the Valsalva maneuver (*p* < 0.05). This increase became more pronounced as the duration of the maneuver was extended, underscoring the differential response of PFO patients under varied physiological testing conditions, especially during prolonged Valsalva maneuvers.

**Conclusion:**

The study confirms the significant value of cTEE in diagnosing PFO under different stimulation states, particularly emphasizing the application of the Valsalva maneuver to significantly improve the sensitivity and specificity of PFO detection. Thus, incorporating cTEE examinations under various stimulation states holds significant clinical importance for enhancing the accuracy and efficiency of PFO diagnosis.

## Graphical abstract

Patients suspected of having patent foramen ovale (PFO) were enrolled to investigate the diagnostic performance of transthoracic echocardiography in different states of stimulation. The results showed that incorporating cTEE examinations under various stimulation states holds significant clinical importance for enhancing the accuracy and efficiency of PFO diagnosis.

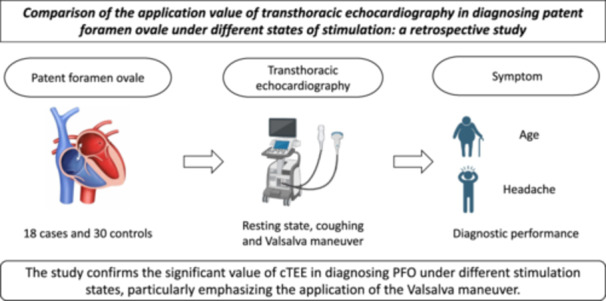

## 1 Introduction

Patent Foramen Ovale (PFO) represents a common cardiac anatomical anomaly present in approximately 25% of adults [1]. While PFO is benign in most instances, it has been associated with certain medical conditions, such as cryptogenic stroke, migraines, and potential thromboembolic diseases [2]. Consequently, the accurate diagnosis of PFO is crucial for assessing the risk of these conditions and formulating appropriate treatment strategies. Contrast‐enhanced transthoracic echocardiography (cTEE) serves as a widely utilized noninvasive diagnostic approach for detecting PFO, involving the use of ultrasound waves and contrast agents to observe cardiac structures and functions [3]. However, the diagnostic process of cTEE can vary depending on the patient's physiological state, such as their state of stimulation. Common stimuli include the resting state, coughing, and the Valsalva maneuver (VM)—a technique involving an attempt to exhale against a closed airway [4]. While cTEE provides baseline data in the resting state without additional stimuli, it may not reveal all instances of PFO. In contrast, the VM, by increasing intrathoracic pressure, significantly enhances the sensitivity of detecting right‐to‐left shunts (RLS) in the diagnosis of PFO [5]. Under this stimulus, cTEE shows greater diagnostic accuracy. A study found that during the VM, a significant percentage of patients exhibited right‐to‐left cardiac shunting in transcranial Doppler (TCD) assessments, while a similar percentage showed shunting in cTEE. These findings underscore the crucial role of the VM in revealing shunts across both diagnostic modalities [6].

This study aims to compare the application value of cTEE in diagnosing PFO under different states of stimulation. By assessing the resting state, coughing, and varying durations of the VM, the research will explore the sensitivity and specificity of PFO detection under these conditions. This investigation holds significant importance for improving the diagnostic methods for PFO, contributing to enhanced diagnostic accuracy and efficiency.

## 2 Materials and Methods

### 2.1 Study Participants

This study consecutively recruited adult patients from October 2022 to February 2024, who exhibited unexplained clinical symptoms such as syncope, headaches, dizziness, and strokes, with a clinical suspicion of PFO. All participants underwent standard transthoracic echocardiography (TTE) and cTTE across three different states of stimulation. Based on the detection of microbubbles in the left atrium within three to six cardiac cycles post‐contrast injection, patients were categorized into the PFO group (microbubbles observed) and the control group (no microbubbles observed) [7]. Patients diagnosed with PFO underwent further evaluation with transesophageal echocardiography (TEE) or underwent a foramen ovale closure procedure. The study groups were divided as follows: (1) resting state, coughing, VM for 5 s, VM for 10 s, and VM for 15 s groups; (2) transthoracic echocardiography group and transesophageal echocardiography group. This is a retrospective study and has received approval from the ethics committee. All patients and some requiring TEE examinations were informed about the purpose, methodology, potential risks, and mitigation measures of the echocardiographic studies and they signed informed consent forms.

### 2.2 Inclusion and Exclusion Criteria

Inclusion criteria: (1) Patients without contraindications to right heart catheterization, able to complete transthoracic and TEE examinations and standard VMs; (2) Patients agreeing to the examinations and signing informed consent forms. Exclusion criteria: (1) Patients with contraindications to right heart catheterization; (2) Patients with poor echocardiographic window, making it difficult to accurately observe shunt bubbles (e.g., patients with chronic obstructive pulmonary disease, morbid obesity); (3) Patients unable to cooperate with the examination or whose family did not agree to participate; (4) Patients with unclear transthoracic echocardiographic views; (5) Patients with structural heart diseases other than PFO; (6) Patients with cognitive impairments or unable to perform VMs and coughing; (7) Patients in the acute phase of infection, with severe heart failure, renal failure, pulmonary failure, or coagulation disorders.

### 2.3 Methodology and Criteria for Judgment

#### 2.3.1 TTE and TEE

Patients were positioned in either the left or right lateral decubitus position for transthoracic and/or transesophageal echocardiographic examinations through right heart contrast imaging. TTE utilized the standard four‐chamber view, while TEE employed the bicameral view to clearly display the foramen ovale flap. The right heart contrast procedure involved the rapid injection of a mixture comprising 8 mL of saline, 1 mL of air, and 1 mL of blood, agitated more than 10 times before rapid administration via the median cubital vein. Echocardiographic imaging storage [8]: Dynamic videos of right heart contrast echocardiography during 15 cardiac cycles were stored at rest, during coughing, and during VMs at 5, 10, and 15 s. Outcome interpretation: Observation and recording of the presence and quantity of microbubbles in the left atrium within six cardiac cycles post‐contrast through video playback.

#### 2.3.2 Transthoracic and Transesophageal Right Heart Contrast Echocardiography

A venous pathway was established in the patient's median cubital vein, connected to a three‐way stopcock; two 10 mL syringes were prepared, one with 8 mL of saline and the other with 1 mL of air, connected via the stopcock. After ensuring patency of the venous pathway and retracting 1 mL of blood, saline, blood, and air were agitated between the two syringes thirty times before evenly paced injection. The apical four‐chamber view was selected to observe the presence and quantity of microbubbles in the left heart after right heart contrast imaging at rest and during the VM for three to five cardiac cycles. The positive quantification criteria for contrast‐enhanced TTE (C‐TTE) followed the method of Calvert et al.: Grade I, < 5 microbubbles in the left heart chamber; Grade II, 5–25 microbubbles; Grade III, > 25 microbubbles but with lower density; Grade IV, > 25 microbubbles, dense, causing opacification of the heart chamber [9]. For C‐TTE, a 20G catheter was placed in the patient's left median cubital or antecubital vein, connected to a three‐way stopcock with two 10 mL syringes attached, filled with 8 mL of saline, 1 mL of air, and 1 mL of the patient's blood [10]. The mixture was agitated at least 20 times to prepare a saline solution rich in microbubbles, which was then rapidly injected during the patient's resting state, coughing, VM, and modified VM (mVM), with at least a 5‐min interval between injections. Observation focused on the cardiac cycle when left heart microbubbles first appeared and the quantity of microbubbles. For transesophageal right heart contrast echocardiography (C‐TEE), if PFO presence was uncertain, the agitation method for saline preparation was identical to that of TTE, with rapid intravenous injection after preparation. The appearance of microbubbles in the left atrium within three cardiac cycles post right heart bubble opacification indicated PFO. This procedure was performed up to three times, with at least a 5‐min interval between attempts [11].

#### 2.3.3 VM

Patients were instructed to pinch their nostrils, close their mouth, and forcefully attempt to exhale, engaging the abdomen without allowing air to escape, and suddenly releasing the breath after 5, 10, and 15 s.

### 2.4 Statistical Methods

Data analysis was performed using SPSS software version 25.0. Count data were presented as case numbers and percentages, with TEE serving as the gold standard for the diagnostic test of C‐TTE. The methods used for intergroup comparisons included the following:

The *χ* ^2^ test was employed to compare the gender distribution differences between the PFO group and the control group. Independent samples *t*‐tests were used to compare age differences between these groups. Analysis of Variance (ANOVA) was conducted to compare the initial appearance time of microbubbles and the quantity of microbubbles under different stimulation states (resting state, coughing, VMs of 5, 10, and 15 s). The *K*‐value was used to compare the distribution differences in microbubble quantities under various stimulation states. Statistical significance (*p* values) was determined to identify significant differences between groups, such as the differences in microbubble quantity and appearance time.

## 3 Results

### 3.1 Clinical Case Data

In this study, we compared the differences between the PFO group and the control group in terms of gender, age, clinical symptoms, and the positivity rate of TTE examinations. The results indicated no significant difference in gender distribution between the two groups (*p* = 0.243). However, there was a statistically significant difference in age between the PFO group and the control group (mean ages were 47.4 ± 14.6 years and 49.4 ± 17.7 years, respectively; *p* = 0.034). Analysis of clinical symptoms revealed that headaches were significantly more prevalent in the PFO group compared to the control group (89.5% vs. 10.5%, *p* < 0.001), while other symptoms such as syncope, dizziness, and cerebral infarction did not show significant differences, although the incidence of cerebral infarction was higher in the PFO group (80% vs. 20%, *p* = 0.05). The positivity rate of TTE examinations was higher in the PFO group both at rest and during coughing, reflecting the correlation between PFO and specific clinical manifestations, as shown in Table 1.

**Table 1:** Clinical and pathological data analysis.

Category	Number of cases	PFO group	Control group	*p* value
Gender				
Male	28	10 (35.7%)	18 (64.3%)	0.243
Female	20	8 (40.0%)	12 (60.0%)	
Age ($\bar{x}$ ± s)		47.4 ± 14.6*	49.4 ± 17.7	0.034
Symptoms				
Syncope	1	1 (100.0%)	0 (0.0%)	0.342
Headache	19	17 (89.5%)*	2 (10.5%)	< 0.001
Dizziness	24	15 (62.5%)	9 (37.5%)	0.342
Stroke	10	8 (80.0%)	2 (20.0%)	0.050
TTE positive examination				
Resting state	7	6 (85.7%)	1 (14.3%)	0.004
Coughing action	11	9 (81.8%)	2 (18.2%)	< 0.001
Valsalva maneuver of different durations				
5 s	12	10 (83.3%)	2 (16.7%)	< 0.001
10 s	16	13 (81.3%)	3 (18.8%)	< 0.001
15 s	21	16 (76.2%)	5 (23.8%)	< 0.001

* Compared with the normal group, *p* < 0.05.

### 3.2 Distribution of Initial Appearance Time of Left Heart Microbubbles Under Different States of Stimulation

In this study, we analyzed the performance of 18 PFO patients under various physiological testing conditions, including at rest, during coughing, and during VMs of varying durations (5, 10, 15 s). The results showed that at rest, 7 PFO patients were identified, with two scoring less than 3 and five falling within the 3–5 range, with no patients scoring above 5. During the coughing test, out of 11 PFO patients, 3 scored less than 3, 7 scored within the 3–5 range, and 1 scored above 5. For VMs of different durations, the distribution of scores among patients performing 10‐ and 15‐s maneuvers indicated that the number of patients scoring above 5 gradually increased (from 0 to 2) as the duration of the maneuver increased (Table 2).

**Table 2:** Distribution of initial appearance of left heart microbubbles in the PFO group under different provocation tests.

	Number of cases	PFO group (total of 18 cases)		
Item		< 3	3–5	> 5
Resting state	7	2	5	0
Coughing action	11	3	7	1
Valsalva maneuver of different durations (5/10/15 s)	10	3	7	0
	13	3	9	1
	16	5	9	2

### 3.3 Comparison of Microbubble Appearance Time Under Different States of Stimulation in CTTE

We explored the differences in the initial cardiac cycle of microbubble appearance indicating RLS under various states of stimulation (resting state, coughing, and VMs of varying durations). The average cardiac cycle at rest was 3.43, during coughing it was 2.86, and during VMs of different durations, the cardiac cycles were 3.17 (5 s), 2.82 (10 s), and 1.54 (15 s), respectively. This demonstrates a significant reduction in cardiac cycles as the duration of the VM increases (*p* < 0.001, indicating a significant difference compared to the resting state) (Table 3).

**Table 3:** Comparison of the initial appearance of RLS microbubbles cardiac cycle under different provocation states.

Category	Number of cases	Cardiac cycle (number)	*p* value
Resting state	7	3.43	
Coughing action	11	2.86**	< 0.01
Valsalva maneuver of different durations	10	3.17 (5 s)*	< 0.05
	13	2.82 (10 s)**	< 0.01
	16	1.54 (15 s)***	< 0.001

* Compared with the resting state, *p* < 0.05.

** Compared with the resting state, *p* < 0.01.

*** Compared with the resting state, *p* < 0.001.

### 3.4 Comparison of RLS Microbubble Quantities Under Different States of Stimulation in TTE

By comparing the quantity of RLS microbubbles at rest, during coughing, and during VMs of varying durations, we observed that at rest, among 7 patients, 57.1% had fewer than 5 microbubbles, 28.6% had between 5 and 10 microbubbles, and 14.3% had between 10 and 30 microbubbles, with no patients exceeding 30 microbubbles. The *K*‐value for this state was 50.32, with *p* < 0.001, indicating statistically significant differences compared to other states. During coughing, among 11 patients, 27.3% had fewer than 5 microbubbles, 36.4% had between 5 and 10, and 18.2% had between 10 and 30 and over 30 microbubbles. The *K*‐value was 37.43, with *p* < 0.001. VMs of varying durations (5, 10, 15 s) showed an increasing trend in the quantity of microbubbles with the duration of the maneuver. For instance, at 15 s, 6.3% of patients had fewer than 5 microbubbles, 18.7% had between 5 and 10, and 37.5% had between 10 and 30 and over 30 microbubbles. The corresponding *K*‐values were 61.32, 65.32, and 71.32, with *p* < 0.001, indicating a significant increase compared to the resting state (Table 4).

**Table 4:** Comparison of RLS microbubble quantity under different provocation methods.

Category	Number of cases	Microbubble quantity				*K*‐value	*p* value
		< 5	5–10	10–30	> 30		
Resting state	7	4 (57.1%)	2 (28.6%)	1 (14.3%)	0	50.32	< 0.001
Coughing action	11	3 (27.3%)	4 (36.4%)	2 (18.2%)	2 (18.2%)	37.43	< 0.001
Valsalva maneuver of different durations	10 (5 s)	1 (10%)	3 (30%)	4 (40%)	2 (20%)	61.32	< 0.001
	13 (10 s)	2 (15.4%)	3 (23.1%)	4 (30.7%)	4 (30.8%)	65.32	< 0.001
	16 (15 s)	1 (6.3%)	3 (18.7%)	6 (37.5%)	6 (37.5%)	71.32	< 0.001

*Note:* Compared with the resting state, *p* < 0.05.

## 4 Discussion

PFO is a common cardiac structural anomaly observed in approximately 25% of adults, which is mostly harmless but has been linked to conditions such as cryptogenic stroke, migraines, and certain types of thromboembolic diseases [12, 13]. Despite the prevalence of PFO, its diagnosis remains challenging, especially in asymptomatic cases. Traditional diagnostic methods include cTEE and TEE, whose effectiveness may be influenced by the patient's state, such as resting, coughing, and performing VMs [14, 15]. This study, by comparing the application value of cTEE in diagnosing PFO under different states of stimulation, revealed the correlation between age and headache symptoms with PFO and highlighted that the significant increase in microbubble quantity during VMs can enhance the sensitivity and specificity of PFO detection. The results underscore the clinical importance of considering cTEE examinations under different states of stimulation to improve the accuracy and efficiency of PFO diagnosis.

Our findings indicate significant differences in age between the PFO and control groups, with a notably higher incidence of headaches in the PFO group. These observations align with the study by Seward et al., which also reported a connection between PFO and migraines [16]. This could be due to PFO allowing microemboli in the blood to bypass pulmonary filtration and directly enter the brain, triggering headaches. Additionally, our study found that VMs significantly increase the sensitivity of PFO detection, consistent with the findings of Brisson et al., who noted that VMs can increase RLS [17], thereby enhancing PFO detection sensitivity. This result emphasizes the importance of incorporating VMs in cTEE examinations. The significant increase in microbubble quantity during VMs and its positive correlation with the duration of the maneuver provides a new perspective for PFO diagnosis. Van Laecke and Pan et al. also reflected this, finding an increase in microbubble quantity positively correlated with the size of PFO, which might facilitate more refined risk stratification in PFO diagnosis [18, 19]. These data reveal the differential response of PFO patients under various physiological tests, particularly during longer durations of VMs, reflecting potential cardiovascular regulatory abnormalities. This variability may have clinical significance for the diagnosis and management of PFO, suggesting that further research is needed to understand the physiological response characteristics of PFO patients more deeply [20].

Our results also indicate that the state of stimulation significantly affects the initial cardiac cycle of RLS microbubble appearance, especially during the VM. With an increase in the duration of the maneuver, there is a significant decrease in cardiac cycles, which may reflect changes in cardiac physiological responses and the sensitivity of RLS detection. This finding is of significant importance for understanding the clinical assessment and diagnosis of RLS, highlighting the necessity to consider changes in cardiac cycles under different states of stimulation.

A major limitation of this study is the relatively small sample size, which might affect the generalizability of the results [21]. Additionally, the retrospective analysis design could introduce selection bias [22]. Future studies should validate these findings through prospective, multicenter research and explore the differential responses of different PFO sizes and shapes under various states of stimulation.

In conclusion, this study highlights the application value of cTEE in diagnosing PFO under different states of stimulation, particularly with the use of VMs. These findings provide important insights for clinical practice regarding PFO diagnosis, aiding in enhancing diagnostic accuracy and efficiency. Further research is needed on a larger sample size to validate these results and explore other factors that may influence PFO diagnosis, to further optimize diagnostic and treatment strategies for PFO.

## Conflicts of Interest

The authors declare no conflicts of interest.

## Data Availability

The data that support the findings of this study are available from the corresponding author upon reasonable request.
